# Cancer-Associated Fibroblast Risk Model for Prediction of Colorectal Carcinoma Prognosis and Therapeutic Responses

**DOI:** 10.1155/2023/3781091

**Published:** 2023-04-25

**Authors:** Yan Wang, Zhengbo Chen, Gang Zhao, Qiang Li

**Affiliations:** ^1^Department of Anesthesiology, State Key Laboratory of Oncology in South China, Sun Yat-sen University Cancer Center, Guangzhou, 510060 Guangdong, China; ^2^Department of Vascular and Plastic Surgery, Guangdong Provincial People's Hospital (Guangdong Academy of Medical Sciences), Southern Medical University, Guangzhou, 510080 Guangdong, China

## Abstract

Colorectal carcinoma (CRC) is a malignant tumor of the digestive system. Cancer-associated fibroblasts (CAFs) are important cellular elements in the tumor microenvironment of CRC, which contribute to CRC progression and immune escape. To predict the survival outcome and therapeutic responses of CRC patients, we identified genes connected with stromal CAF and generated a risk model. In this study, we used multiple algorithms to reveal CAF-related genes in the Gene Expression Omnibus and The Cancer Genome Atlas datasets and construct a risk model composed by prognostic CAF-associated genes. Then, we evaluated whether the risk score could predict CAF infiltrations and immunotherapy in CRC and confirmed the expression of the risk model in CAFs. Our results showed that CRC patients with high CAF infiltrations and stromal score had worse prognosis than those with low-CAF infiltrations and stromal score. We obtained 88 stromal CAF-associated hub-genes and generated a CAF risk model consisting of ZNF532 and COLEC12. Compared with low-risk group, the overall survival in high-risk group was shorter. The relationship between risk score, ZNF532 and COLEC12, and stromal CAF infiltrations and CAF markers was positive. In addition, the effect of immunotherapy in the high-risk group was not as good as that in the low-risk group. Patients with the high-risk group were enriched in chemokine signaling pathway, cytokine-cytokine receptor interaction, and focal adhesion. Finally, we confirmed that the expressions of ZNF532 and COLEC12 in risk model were widely distributed in fibroblasts of CRC, and the expression levels were higher in fibroblasts than CRC cells. In conclusion, the prognostic CAF signature of ZNF532 and COLEC12 can be applied not only to predict the prognosis of CRC patients but also to evaluate the immunotherapy response in CRC patients, and these findings provide the possibility for further development of individualized treatment for CRC.

## 1. Introduction

As a common malignant tumor, the risk of colorectal carcinoma (CRC) is related to individual characteristics or habits such as age, history of chronic diseases, and lifestyle [1]. Currently, there are various screening methods for CRC, such as colonoscopy, fecal occult blood test, multitarget stool DNA test, and fecal immunochemical test [2]. Although early screening can improve the curability of CRC, it is necessary to improve the screening methods and accuracy of CRC because of its slow growth and easy to be confused with other cancers [3, 4]. As a consequence, identification new markers of tumor metastasis are important for improving CRC diagnosis and prognosis.

Tumor microenvironment is the cellular environment in which cancer cells exist, including fibroblasts, immune cells, and extracellular matrix (ECM) [5]. The acquisition and maintenance of tumor markers depend on the role of tumor microenvironment to varying degrees. There are a large number of tumor-associated fibroblasts in the tumor microenvironment, which actively participate in cancer progression through complex interactions with other cell types [6]. Clinically, cancer-associated fibroblast (CAF) markers are associated with poor prognosis in many types of cancer [7]. Researchers have identified the heterogeneity of CAFs in breast cancer by single-cell RNA sequencing, and the identification of CAF-specific markers provides support for the development of drugs targeting CAFs [8]. Nowadays, CAF-derived key genes have diagnostic efficacy for gastric cancer [9]. Factors secreted by CAFs can promote the progression of CRC [10]. However, whether stromal CAF-related gene expression signatures could predict clinical outcomes of CRC remains unknown.

Herein, we collected stromal CAF-related factors datasets from the Gene Expression Omnibus (GEO) and The Cancer Genome Atlas (TCGA) databases. Next, we performed weighted gene coexpression network analysis (WGCNA) for identifying the hub-genes of stromal CAFs and construct a risk model composed ZNF532 and COLEC12 by univariate and the least absolute shrinkage and selection operator (LASSO) Cox regression to predict CRC prognosis and treatment effects. Our results offer new markers and therapeutic approaches for the diagnosis and prognosis of CRC.

## 2. Materials and Methods

### 2.1. Data Acquisition and Preprocessing

The microarray and clinical data were obtained from GEO database (GSE39582, 566 tumor samples). The RNA sequence and single-nucleotide polymorphism (SNP) mutation data were obtained from TCGA COAD database (TCGA-COAD, 476 tumor samples). We picked GSE39582 as the training cohort, and TCGA-COAD was chosen for validation cohort. Additionally, the single-cell RNA sequencing data was obtained from GSE132465 from the GEO database.

### 2.2. CAF and Stromal Score Calculation, Survival Analysis

According to other reports [11–14], “EPIC,” “MCPcounter” and “xCell” R packages, and “TIDE” algorithm (http://tide.dfci.harvard.edu/) were applied to evaluate CAF abundances in tumor samples. The “estimate” package was utilized to evaluate the stromal score. Survival analysis of tumor patients was using “survminer” R package based on CAF and stromal scores.

### 2.3. WGCNA

According to previous report [9], the weighted coexpression analysis was performed using the “WGCNA” package to analyze the coexpression modules associated with CAF and stroma. The hub-genes were selected from the most relevant modules according to the threshold criteria (module membership > 0.8 and gene significance > 0.4) (Figure S1A and B).

### 2.4. Functional Enrichment Analysis of Hub-Genes

The “clusterProfiler” package was utilized to analyze functional enrichment information of hub-genes. The graphics were drawn using “enrichplot” package.

### 2.5. Risk Model Construction and Validation

Univariate Cox analysis was utilized to identify prognostic genes. Next, the risk model was built by LASSO Cox regression analysis. Then, the patient's risk score was calculated. The “survminer” R package was utilized to analyze the survival outcomes of different risk groups.

### 2.6. Association Analysis between Risk Score and CAF Score

The Cor function was utilized to calculate the correlation between risk score and CAF score, and the “GGally” package was utilized to analyze the pairwise correlation map. The “Pheatmap” package was utilized to analyze the cluster maps of risk genes and CAF known marker genes. The Cor function was utilized to calculate the relationship between risk genes, risk scores, and CAF marker genes.

### 2.7. Immunotherapy Prediction

The effect of each sample tumor immunotherapy was predicted by the TIDE algorithm. The pROC package was utilized to identify the accuracy of the model's predictions.

### 2.8. Gene Set Enrichment Analysis (GSEA)

The GSEA was utilized to analyze the pathways enriched in different risk groups by the “clusterProfiler” package.

### 2.9. SNP Analysis

The “Maftools” package was applied to analyze high- and low-risk mutant genes, mutation types, and maps waterfalls and then compare whether there was a difference in tumor mutation burden (TMB) between the high- and low-risk groups.

### 2.10. Single-Cell RNA Sequencing Analysis

In this study, the CRC single-cell sequencing dataset was obtained from the GEO database (GSE132465, 10 normal samples and 23 tumor samples). The effect of cell cycle on subsequent results was removed using the SCTransform function. A standardized “SCT” method was used to integrate different samples to eliminate batch effects. Cells were reduced in dimension by principal component analysis (PCA), and then, cell clustering was displayed by uniform manifold approximation and projection (UMAP) method. Cells were then annotated by BlueprintEncodeData dataset and known cell markers in the singleR and celldex packages. The gene set variation analysis (GSVA) R package was used to assess potential changes in pathway activity in each CAF subcluster.

### 2.11. Validation of ZNF532 and COLEC12 Expression on CAFs

The mRNA expressions of the Cancer Cell Line Encyclopedia (CCLE) database were used to analyze the expression of ZNF532 and COLEC12 in fibroblasts and CRC cells. Human colorectal fibroblast CCD-18-co was purchased from ATCC (Manassas, UAS), and SW480 cells were provided by the Shanghai Academy of Biological Sciences. Cells were cultured in DMEM medium with 10% fetal bovine serum. Total RNA was extracted by TRIzol reagent (Invitrogen, USA). Then, cDNA was prepared using the PrimeScript RT kit (Takara, Nanjing, China). AceQ Universal SYBR qPCR Master Mix (Vazyme, Nanjing, China) was used on an ABI StepOnePlus™ real-time quantitative PCR (q-PCR) instrument (Applied Biosystems, CA, USA) for q-PCR. Primer information was given in Table S1. GAPDH was the internal parameters of q-PCR.

### 2.12. Statistical Analysis

The overall survival (OS) of high- and low-risk groups was displayed by Kaplan–Meier curves. GraphPad Prism 8.0 was performed for statistical analyses. Student's *t*-test was used for comparison between two groups. Statistical significance was regarded as *p* values < 0.05.

## 3. Results

### 3.1. Higher CAF Infiltrations and Stromal Scores Had Poor OS in Patients with CRC

**Figure 1:**
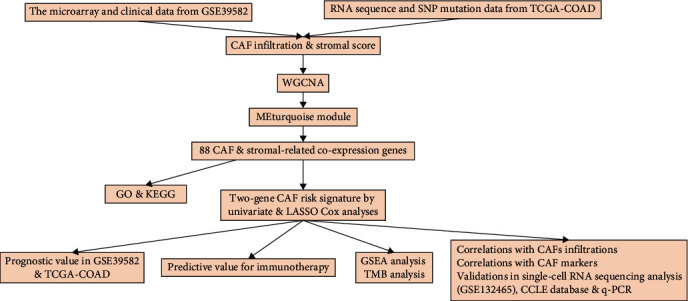
The schematic diagram of the workflow.

**Figure 2:**
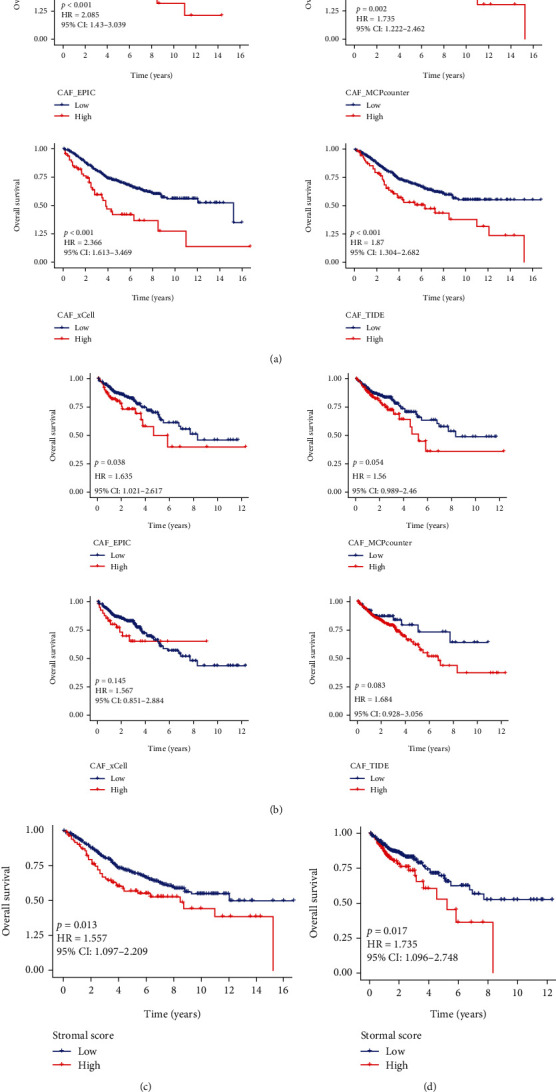
High CAF and stromal scores in CRC had a bad prognosis. High CAF immune infiltration level was associated with poor prognosis in GSE39582 (a) and TCGA-COAD (b) cohorts. High stromal score was associated with poor prognosis in GSE39582 (c) and TCGA-COAD (d) cohorts.

Figure 1 displayed the work chart of our study. We used the EPIC, MCP-counter, xCell, and TIDE methods to evaluate the infiltration of CAFs in tumor microenvironment, and the stromal score was a displayed estimate algorithm. Subsequently, the prognostic values of CAFs on CRC were predicted by Kaplan-Meier curves. As depicted in Figure 2(a), high CAF infiltrations had a shorter OS in patients with CRC in GSE39582 cohort compared with low CAF infiltrations. Similar results were obtained in TCGA-COAD (Figure 2(b)). Additionally, the prognosis of CRC patients with high stromal score was bad both in GSE39582 and TCGA-COAD cohorts (Figures 2(c) and 2(d)). Collectively, the above information highlighted the importance of the relationship between CAF infiltration and CRC prognosis.

### 3.2. WGCNA Analysis Performed for Identifying the Hub-Genes of CAFs

**Figure 3:**
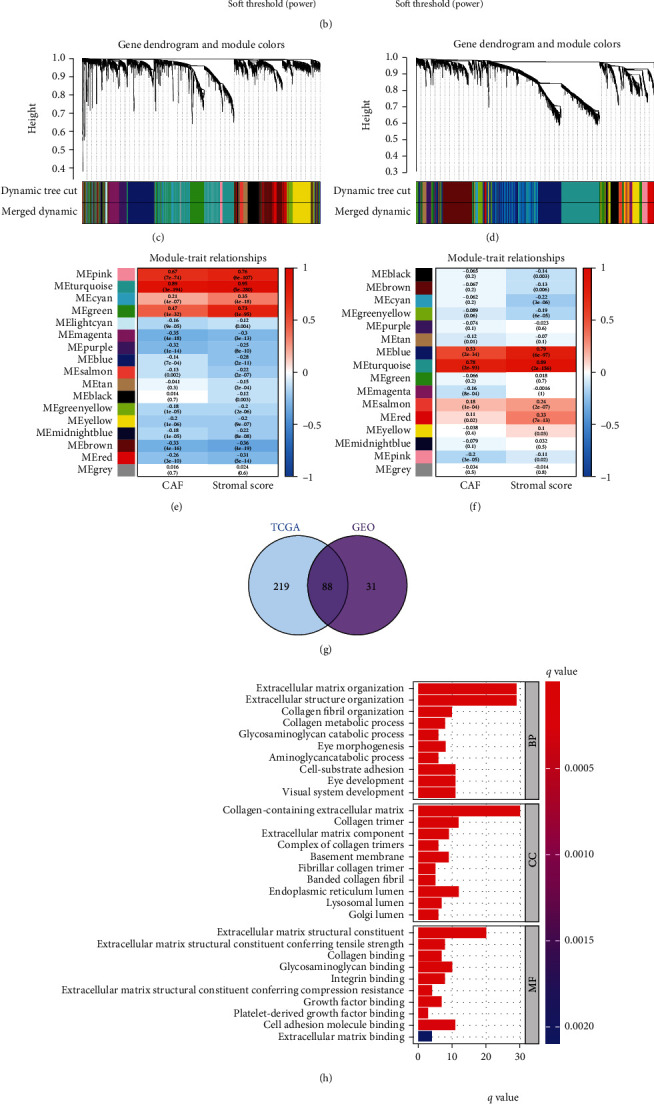
WGCNA was used to explore stromal CAF-related hub-genes and perform functional enrichment analysis. The soft-thresholding power in GSE39582 (a) and TCGA-COAD (b) cohorts. Clustering dendrograms exhibiting hub-genes with alike expression profiles were converged into coexpression modules in GSE39582 (c) and TCGA-COAD (d) cohorts. MEturquoise module was most closely connected with the CAF proportion and stromal score in GSE39582 (e) and TCGA-COAD (f) cohorts. (g) Venn diagram showed the shared hub-genes in GSE39582 and TCGA-COAD cohorts. (h) GO enrichment analysis of 88 shared hub-genes. (i) KEGG enrichment analysis of 88 shared hub-genes.

To filtrate the key genes related to stromal CAFs, we performed WGCNA analysis. We used the soft threshold power of 5 in GSE39582 (Figure 3(a)) and 6 in TCGA-COAD (Figure 3(b)) to construct a scale-free topology network. For GSE39582, 17 coexpression models were clustered by hierarchical clustering tree (Figure 3(c)), and the MEturquoise module was significantly positively associated with the CAF proportion (Correlation = 0.89, *p* = 3*e* − 194) and stromal score (Correlation = 0.95, *p* = 5*e* − 280) (Figure 3(e)). There were 16 coexpression models in TCGA-COAD (Figure 3(d)), in which the MEturquoise module was positively associated with the CAF proportion (Correlation = 0.78, *p* = 2*e* − 93) and stromal score (Correlation = 0.89, *p* = 2*e* − 156) (Figure 3(f)). Thus, a total of 119 and 307 hub-genes, which have the highest correlation with CAF and stromal scores, were screened out in the MEturquoise module of GSE39582 and TCGA-COAD, respectively.

### 3.3. Functional Enrichment Analysis

Eighty-eight hub-genes were acquired by taking the intersection of 2 hub-gene sets from GSE39582 and TCGA-COAD (Figure 3(g)). Subsequently, we performed functional enrichment analysis of these common hub-genes. Gene Ontology (GO) term analysis demonstrated that “extracellular matrix organization,” “collagen-containing extracellular matrix,” and “extracellular matrix structural constituent” were the noteworthy terms in biological process (BP), cellular component (CC), and molecular function (MF), respectively (Figure 3(h)). Moreover, Kyoto Encyclopedia of Genes and Genomes (KEGG) pathways exhibited that these common hub-genes were mainly focused on “ECM-receptor interaction” and “PI3K-Akt signaling pathway” (Figure 3(i)). Studies have shown that the ECM acts as a physical barrier that contributes to cancer cell invasion, inhibits the infiltration of antitumor immune cells, and ultimately promotes tumor deterioration and treatment resistance [5, 15]. In addition, the PI3K-Akt signaling pathway promotes tumorigenesis by regulating cell metabolic reprogramming and invasion and metastasis [16]. Together, these results indicated that these hub-genes have a correlation with tumor progression and immune escape.

### 3.4. Generation of a Stromal CAF-Related Gene (CAFG) Predictive Model

**Figure 4:**
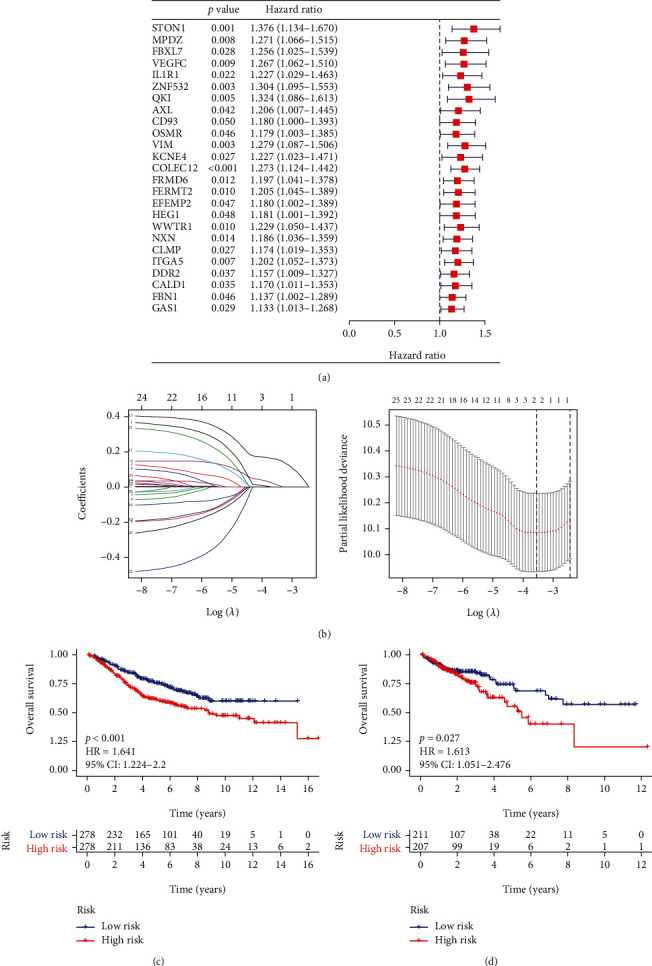
Construction of the prognostic model. (a) Univariate Cox analysis. (b) LASSO Cox regression analysis. (c) Survival analysis in GSE39582 cohort. (d) Survival analysis in TCGA-COAD cohort.

First, univariate Cox regression analysis was performed to study the relationship between the 88 hub-genes and prognosis and obtained that 25 prognostic hub-genes were finally selected in GSE39582 (Figure 4(a)). Next, LASSO Cox analysis was utilized to generate a risk model with 2 genes (ZNF532 and COLEC12) (Figure 4(b)). Then, we figured the risk score as follows: risk score = expression of ZNF532^∗^ 0.017205958 + expression of COLEC12^∗^ 0.158665214. The CRC patients were separated into high- and low-risk groups depending on the median risk score. The OS of patients in the high-risk group was shorter in the GSE39582 cohort than that of low-risk group (*p* < 0.001; Figure 4(c)). This is equally true of TCGA-COAD cohort (*p* = 0.027; Figure 4(d)). These results suggested that the signature of stromal CAFGs was as prognostic marker in CRC.

### 3.5. Stromal CAFGs Have a Strong Correlation with CAF Infiltrations and CAF Markers

**Figure 5:**
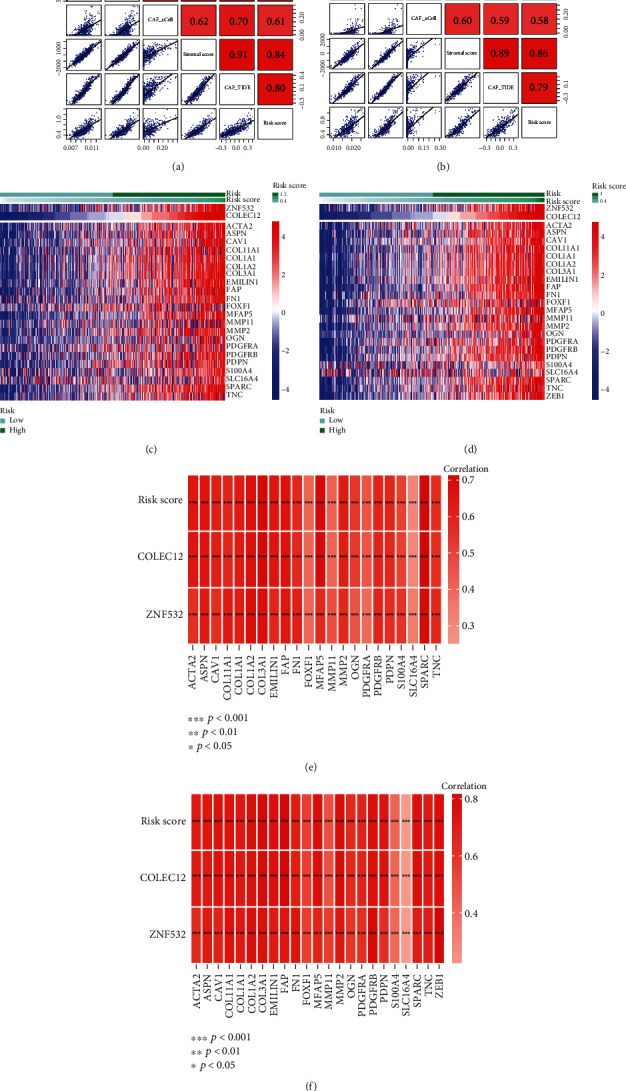
Risk score was positively connected with CAF infiltrations and CAF markers. Risk score was positively associated with CAF abundances in GSE39582 (a) and TCGA-COAD (b) cohorts. CAF markers, ZNF532 and COLEC12, were highly expressed in high-risk group, both in GSE39582 (c) and TCGA-COAD (d) cohorts. CAF markers were positively connected with risk score, ZNF532, and COLEC12 in GSE39582 (e) and TCGA-COAD (f) cohorts.

To further verify whether our CAF model could predict CAF infiltration, we performed Spearman's correlation analyses. As depicted in Figure 5(a), the risk score was significantly positively associated with the CAF infiltrations and stromal score in GSE39582 cohort, which was similar to those in TCGA-COAD cohort (Figure 5(b)). Meanwhile, the expressions of CAF markers in high-risk group were increased comparing to low-risk group both in GSE39582 (Figure 5(c)) and TCGA-COAD (Figure 5(d)) cohorts. Furthermore, all CAF markers were positively associated with the risk core, ZNF532, and COLEC12 in GSE39582 (*p* < 0.001; Figure 5(e)), as well as in TCGA-COAD cohort (*p* < 0.001; Figure 5(f)). Overall, the predictive model composed of ZNF532 and COLEC12 may predict the state of CAF infiltrations in tumor microenvironment.

### 3.6. The Relationship between Risk Score and Immunotherapy

**Figure 6:**
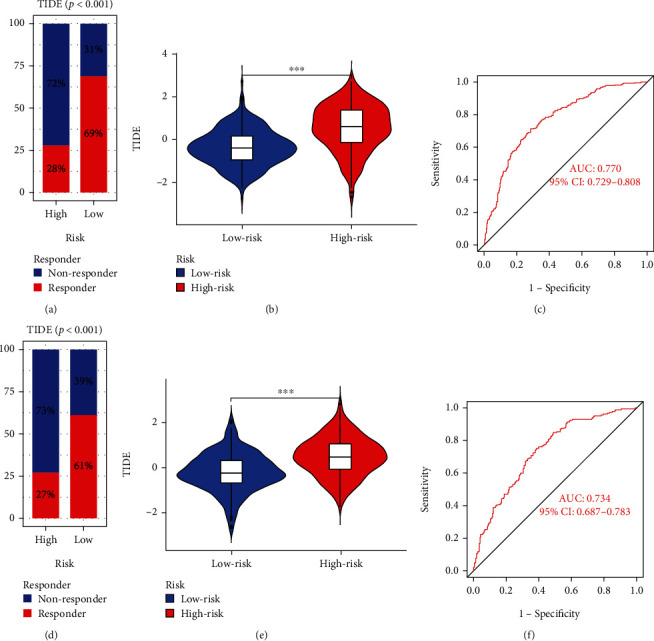
Multidimensional validation for risk score. Comparison of the effect of immunotherapy between the high- and low-risk groups in GSE39582 (a) and TCGA-COAD (d) cohorts. Comparison of the TIDE level between the high-and low-risk groups in GSE39582 (b) and TCGA-COAD (e) cohorts. Receiver-operating characteristic curves of the risk score in forecasting treatment effects in GSE39582 (c) and TCGA-COAD (f) cohorts.

Due to the complexity of tumor immune microenvironment, the effect of immunotherapy in CRC patients is relatively poor [17]. Therefore, we further evaluated whether the risk score could be used as a predictor of immunotherapy in CRC patients. For GSE84437, the high-risk group (28%) was less sensitive to immunotherapy than low-risk group (69%) (*p* < 0.001; Figure 6(a)); compared with low-risk group, the CAF score was elevated in the high-risk group (Figure 6(b)); the area under curve (AUC) value of rick score was 0.770 (95% CI: 0.729–0.808) (Figure 6(c)). For TCGA-COAD, these results were the same as for GEO (Figures 6(d)–6(f)). Briefly, our prognostic model has predictive power for immunotherapy of CRC.

### 3.7. GSEA Enrichment Analysis

**Figure 7:**
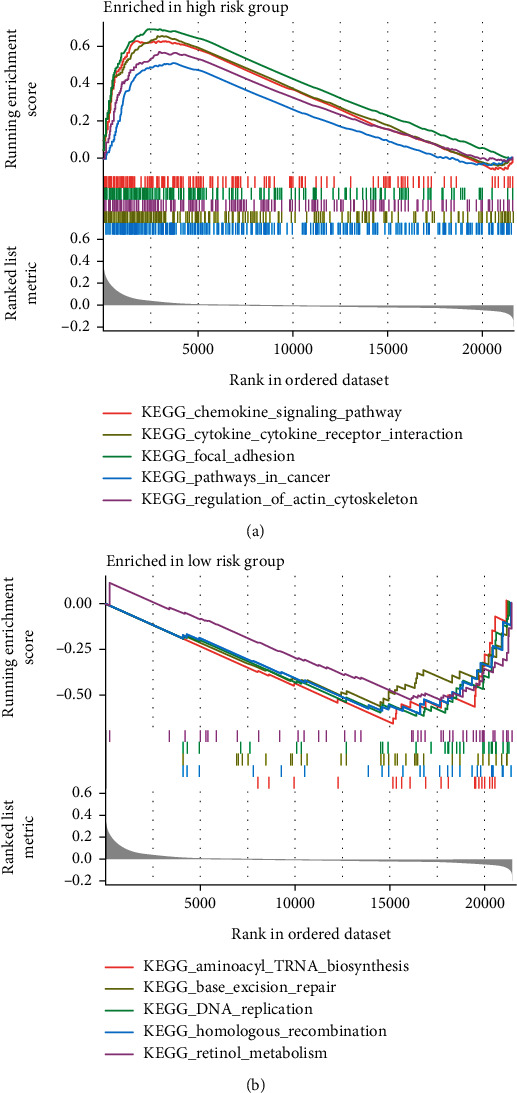
GSEA showing possible associations between high- (a) and low-risk (b) groups and disease phenotypes.

As shown in Figure 7(a), the high-risk group was mainly focused on cytokine-cytokine receptor interaction, chemokine signaling pathway, and focal adhesion. The low-risk group was mainly focused on aminoacyl tRNA biosynthesis, DNA replication, and retinol metabolism (Figure 7(b)).

### 3.8. Correlation between Risk Score and TMB

**Figure 8:**
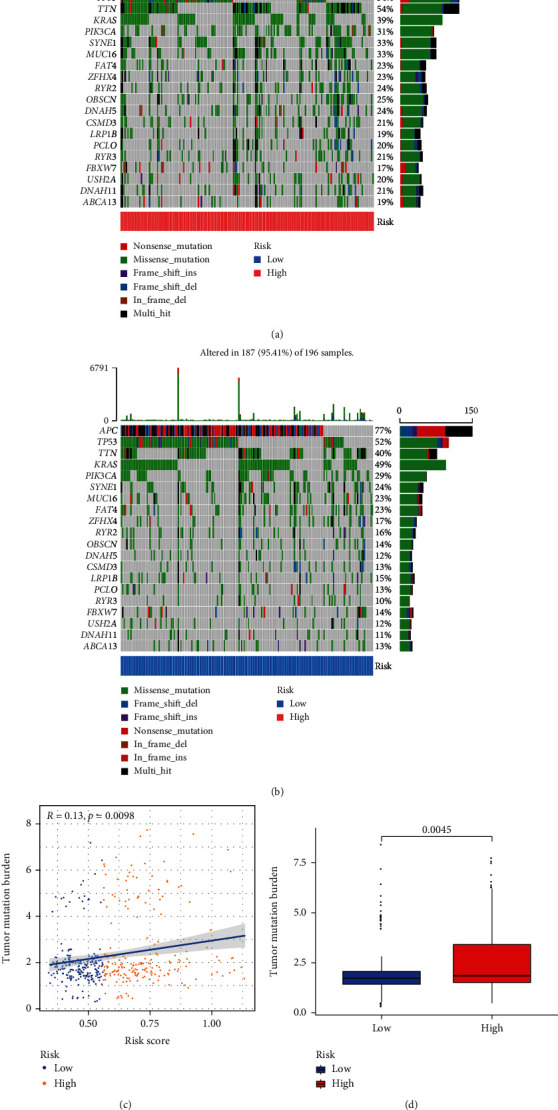
SNP analysis. The top 20 mutational genes in high- (a) and low-risk (b) groups. (c) Analysis of correlation between TMB and the risk groups. (d) Comparison of TMB value between the high- and low-risk groups.

The waterfall plots have displayed top 20 genes with the highest mutational frequencies in the high- (Figure 8(a)) and low-risk (Figure 8(b)) groups, respectively. Surprisingly, these continual mutational genes were shared in the two risk groups. Besides, the risk score has a positive correlation with the TMB value (correlation = 0.13, *p* = 0.0098, Figure 8(c)). Meanwhile, the TMB values were upregulated in the high-risk group compared with low-risk group (*p* = 0.0045; Figure 8(d)). Thus, patients in the high-risk group may benefit more from immune microenvironment with high TMB.

### 3.9. ZNF532 and COLEC12 Identified in Single-Cell Gene Expression Patterns of Fibroblasts

**Figure 9:**
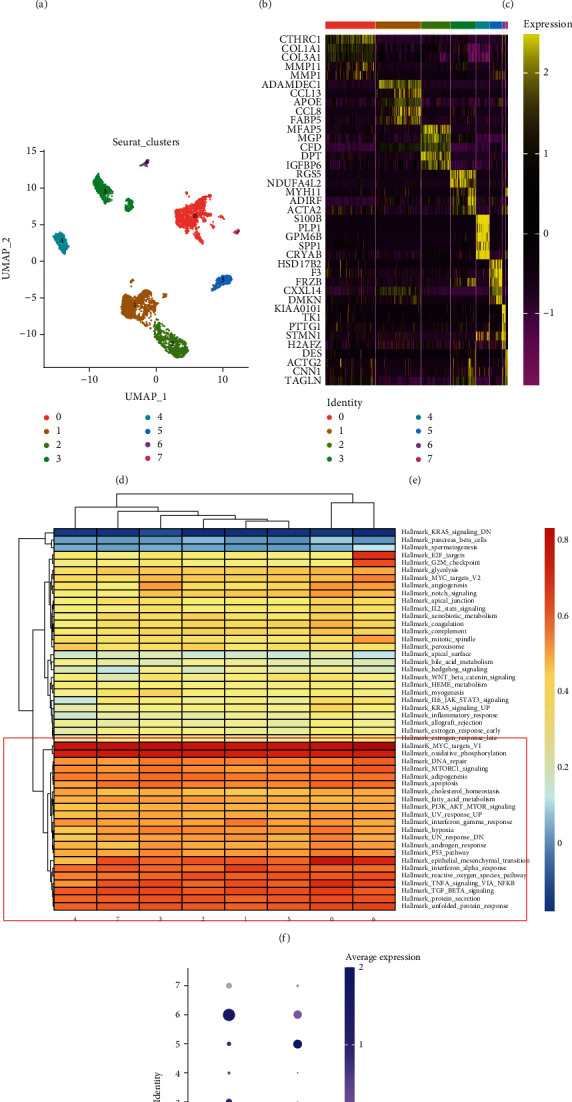
Single-cell RNA sequencing analysis of CRC. UMAP map of cell clusters (a) and types (b). (c) Distribution of ZNF532 and COLEC12 in each cell type. (d) Fibroblasts were divided into 8 subpopulations. (e) The expression of top 5 genes in each fibroblast subpopulation. (f) GSVA analysis of fibroblast subpopulations. (g) Distribution of ZNF532 and COLEC12 in each fibroblast subpopulation.

To describe the expression of ZNF532 and COLEC12 at fibroblasts, we collected single-cell RNA sequencing data from patients with CRC. After preliminary quality control confirmation, 62,716 cells can be used for subsequent analysis. As shown in Figure 9(a), there were 23 kinds of cell clusters in CRC patients, which were mainly divided into B cells, CD4+/8 + T cells, dendritic cells (DC), fibroblasts, mast cells, endothelial cells, macrophages, epithelial cells, monocytes, and plasma cells (Figure 9(b)), according to the expression level of marker genes (Figure S2). Not surprisingly, ZNF532 and COLEC12 were highly expressed in fibroblasts (Figure 9(c)). In addition, ZNF532 was distributed in endothelial cells, while COLEC12 also belonged to macrophage, speculating CAF signature affecting tumor progression by regulating tumor matrix formation and immune infiltration of CRC. Next, we explored the expression of ZNF532 and COLEC12 in fibroblast subpopulations. There were 8 subpopulations of fibroblasts (Figure 9(d)), including cluster 0 (high expressed genes: CTHRC1 and COL1A1), cluster 1 (CCL13), cluster 2 (MGP), cluster 3 (NDUFA4L2), cluster 4 (PLP1), cluster 5 (FRZB), cluster 6 (TK1), and cluster 7 (ACTG2) (Figure 9(e)). GSVA analysis showed that clusters 5 and 6 were mainly enriched in pathways regulating the tumor microenvironment, such as oxidative phosphorylation, TNF-*α* signaling via NF-K*β*, and endothelial-mesenchymal transition (Figure 9(f)). Furthermore, ZNF532 was mainly distributed in cluster 6, and COLEC12 was mainly distributed in cluster 5 (Figure 9(g)), which that suggested cluster 6 and cluster 5 in fibroblasts were mainly involved in tumor progression and immunotherapy of CRC.

### 3.10. Validation of ZNF532 and COLEC12 in Fibroblasts and CRC Cells

**Figure 10:**
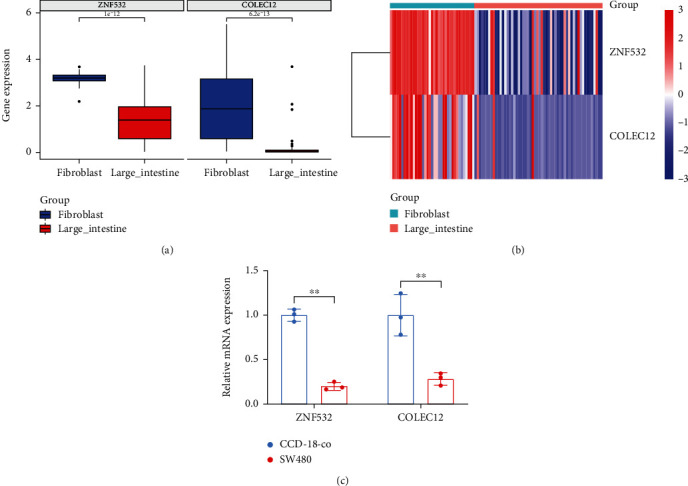
Multidimensional expression validation. The levels of ZNF532 and COLEC12 in the fibroblasts and large intestine were compared by Wilcoxon analysis (a) and exhibited in the heat map (b). (c) q-PCR was applied to verify the expression of ZNF532 and COLEC12 in fibroblasts and SW480. ^∗∗^*p* < 0.01.

Both ZNF532 and COLEC12 were highly expressed in fibroblasts compared to large intestine (Figures 10(a) and 10(b)). To further validate this result, we performed q-PCR validation. Consistently, the mRNA expressions of ZNF532 and COLEC12 were highly expressed in fibroblasts than those in CRC cell line (SW480) (Figure 10(c)). These results indicated that ZNF532 and COLEC12 might be CAF-specific markers.

## 4. Discussion

CRC, as the third cancer incidence rate in worldwide, has yet to be successfully and completely treated with multiple therapeutic options [18, 19]. In the tumor microenvironment, CAFs were the most abundant stromal cells, which regulated the malignant progression and immunotherapy resistance of CRC by secreting cytokines to control cell proliferation and ECM deposition and remodeling [15, 20]. However, limited studies have investigated the function of stromal CAF-related factors on CRC. Here, we found that high levels of CAF and stromal score lead to poor prognosis in CRC. Subsequently, we generated a prognostic CAF model including 2 genes (ZNF532 and COLEC12). Patients in the high-risk group in this model had shorter OS, low sensitivity to immunotherapy, and high levels of TMB. Besides, the risk genes were high expressed in fibroblasts.

CAFs are the major cellular component of desmoplastic stroma characteristic that contribute to tumor progression and immune escape [21]. Consistently, we confirmed that higher CAF and stroma scores were interrelated with worse prognosis in CRC. However, whether CAFGs could be as novel treatment targets in CRC is still unknown. Studies have reported that risk signature composed CAF-secreted cytokines can predict the clinical prognosis of breast cancer patients [22]. CAF-related genes had great and prognostic value for hepatocellular carcinoma prognosis [23]. Consistently, we constructed a CAFG prognostic model of CRC by applying WGCNA and univariate and LASSO Cox regression methods. Based the risk score of each patient, CRC patients with high-risk scores had OS survival than CRC patients with low-risk scores. The signature of high CAF score with poor OS can be used to predict the prognosis of patients with gastric cancer [9]. In view of this, our CAF model had good value for applying to predict CRC prognostic.

In addition to the interaction between CAF and cancer cells, the intricate crosstalk between CAFs and tumor immune microenvironment (TIME) is also the key to promote tumor progression [24]. Infiltrated CAFs interact with other immune cells in TIME to promote the formation of immunosuppressive tumor microenvironment, thereby allowing cancer cells to evade the surveillance of the immune system [25]. In the risk model, CAF abundances in tumor microenvironment were positively connected with the risk score and the levels of ZNF532 and COLEC12. Besides, patients with high-risk scores had lower sensitivity to immunotherapy than patients with low-risk scores. Referring to other similar studies, this means that our CAF risk score has an important predictive effect on immune infiltration of CAF and may regulate the formation of immunosuppressive tumor microenvironment [26]. Meanwhile, the levels of ZNF532 and COLEC12 were increased in fibroblasts compared to CRC cells. These results indicated that ZNF532 and COLEC12 can be CAF-specific markers for CRC, and the CAF risk model can evaluate the level of CAF infiltration in tumor microenvironment.

With respect to ZNF532 and COLEC12 in the model, elevated expression of COLEC12 had a worse prognostic outcome and increased inflammation in osteosarcoma [27]. Moreover, COLEC12 as a cancer stemness-related signature could predict colon adenocarcinoma prognosis [28]. At an epithelial cellular level, activation of ZNF532 could promote the epithelial-to-mesenchymal transition in laryngeal squamous cell carcinoma cells [29]. We observed that COLEC12 and ZNF532 were highly expressed in macrophages and endothelial cells, respectively, which was consistent with the findings that COLEC12 expression was correlated with immune-related molecules [30], and ZNF532 altered the biological activity of endothelial cells [31]. However, their function in CAFs of CRC remains unclear, so further studies of the mechanisms of these CAF markers are needed to explore the progression, resistance, and immunosuppression of CRC.

## 5. Conclusion

In conclusion, higher infiltration of stromal CAFs in tumor microenvironment was associated with poor prognosis in CRC, and ZNF532 and COLEC12 could be as novel prognostic CAF biomarkers by producing the prediction model. Our CAF prediction model could forecast CRC prognosis, CAF infiltrations, and treatment effects, which might offer new targets and potential treatment strategies of CRC.

## Data Availability

The datasets analyzed in this study could be found in GSE39582, TCGA-COAD, and GSE132465.

## Supplementary Materials

Supplementary 1 Figure S1: scatter plots of the MEturquoise module in GSE39582 (A) and TCGA-COAD (B).

Supplementary 2 Figure S2: UMAP plots of markers for each cell type in CRC.

Supplementary 3 Supplementary Table 1: the primer information of genes.
