# Supplementary material for: Cancer-Associated Fibroblast Risk Model for Prediction of Colorectal Carcinoma Prognosis and Therapeutic Responses
Source: Mediators Inflamm. 2023 Apr 25;2023:3781091. doi: 10.1155/2023/3781091 (PMC10154103; doi:10.1155/2023/3781091)
Supplement: Supplementary 1 — Figure S1: scatter plots of the MEturquoise module in GSE39582 (A) and TCGA-COAD (B). [file 3781091.f1.pdf]

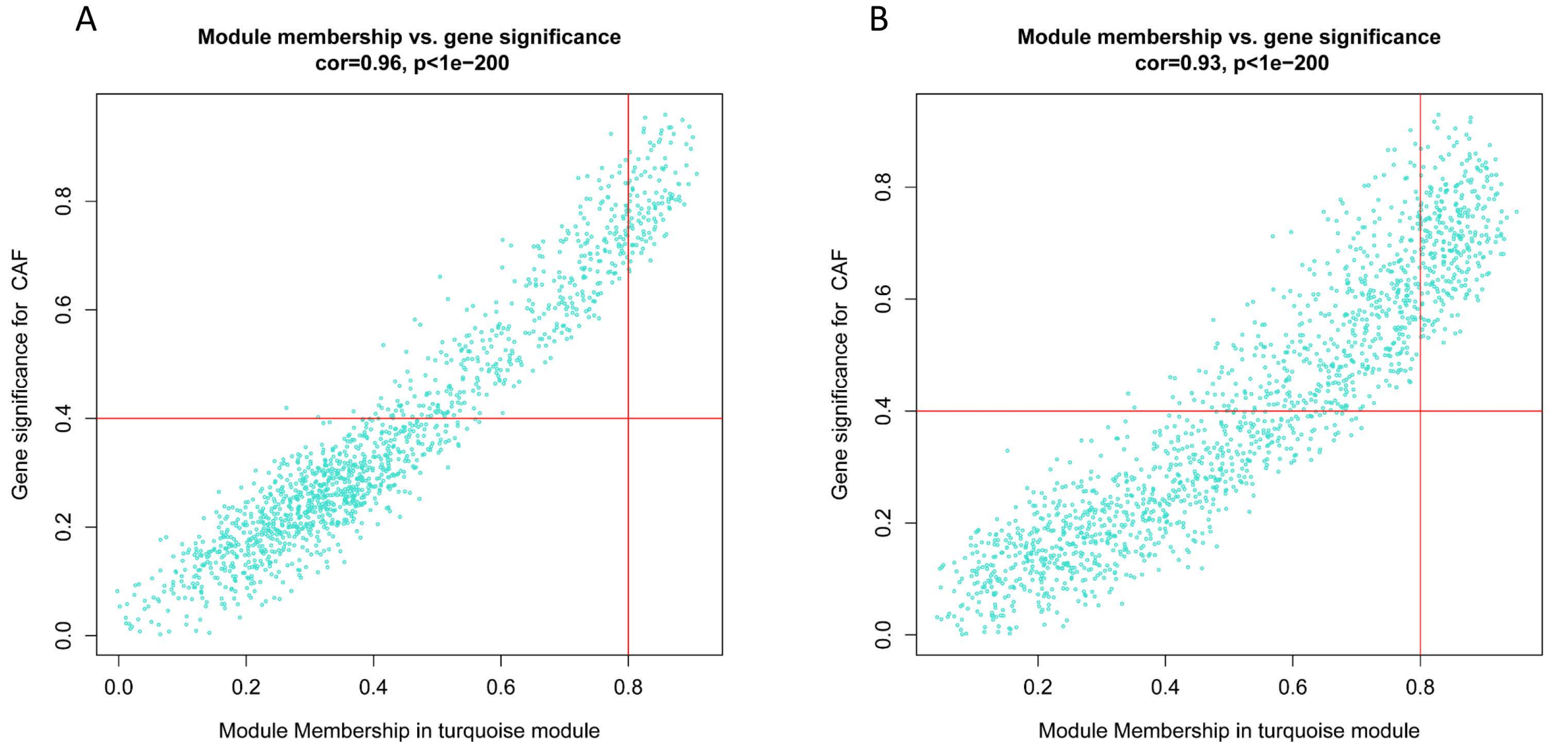
A
Module membership vs. gene significance
cor=0.96, p<1e−200
Gene significance for CAF
0.0
0.2
0.4
0.6
0.8
Module Membership in turquoise module
0.0
0.2
0.4
0.6
0.8
B
Module membership vs. gene significance
cor=0.93, p<1e−200
Gene significance for CAF
0.0
0.2
0.4
0.6
0.8
Module Membership in turquoise module
0.2
0.4
0.6
0.8
