# Supplementary figures and images for: Cancer-Associated Fibroblast Risk Model for Prediction of Colorectal Carcinoma Prognosis and Therapeutic Responses
Source: Mediators Inflamm. 2023 Apr 25;2023:3781091. doi: 10.1155/2023/3781091 (PMC10154103; doi:10.1155/2023/3781091)

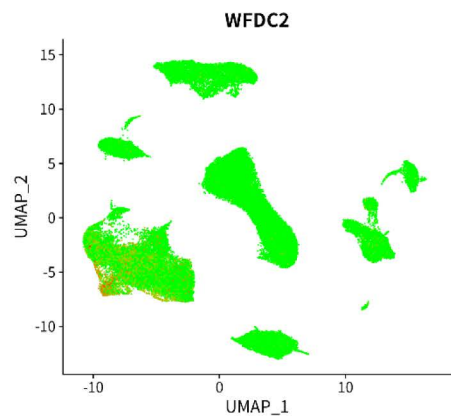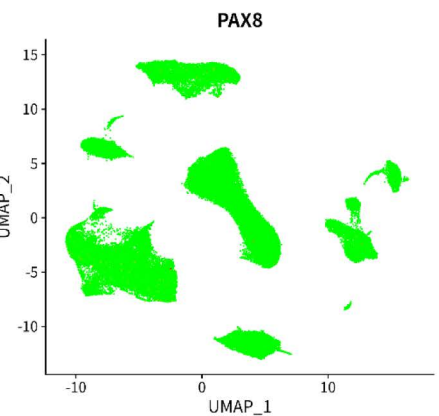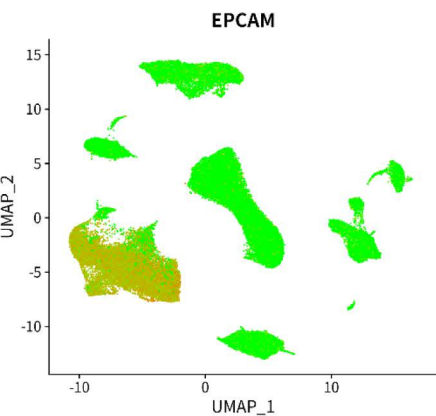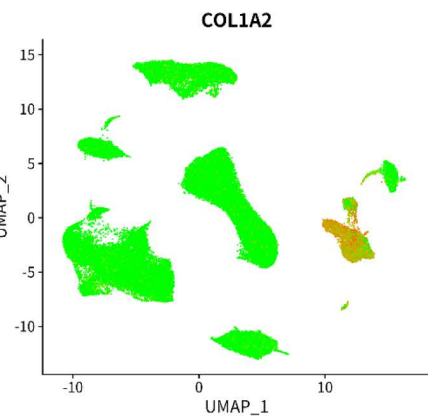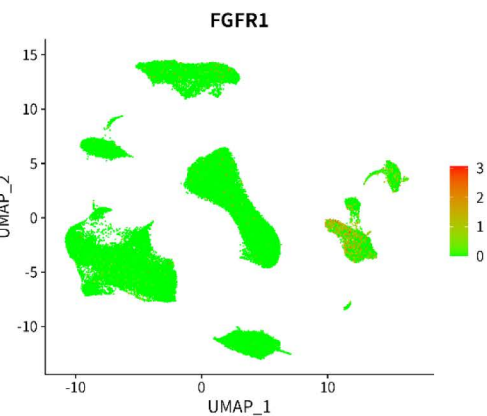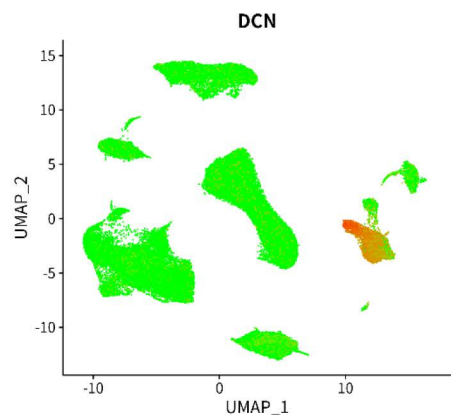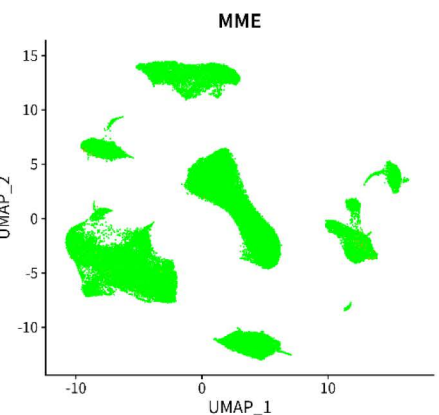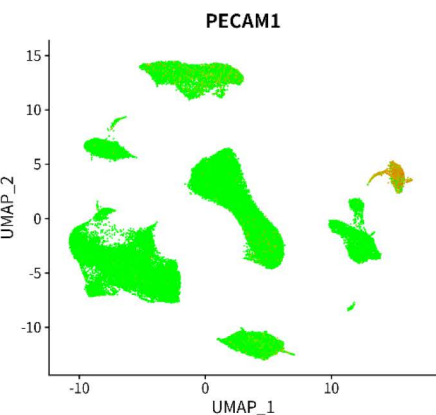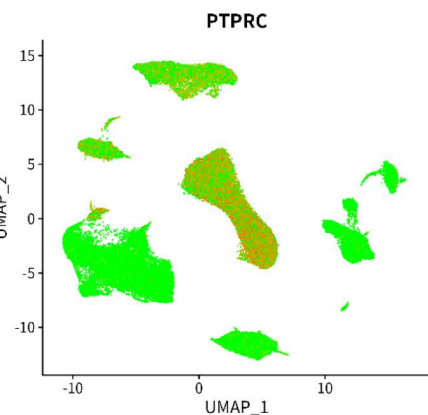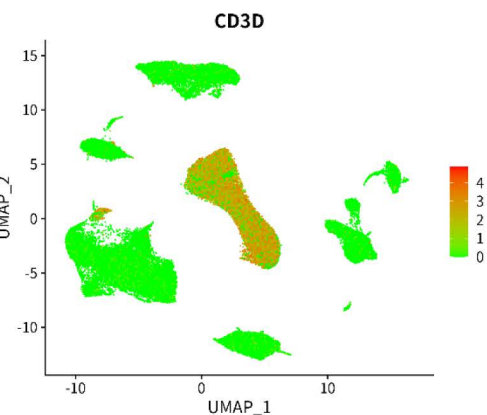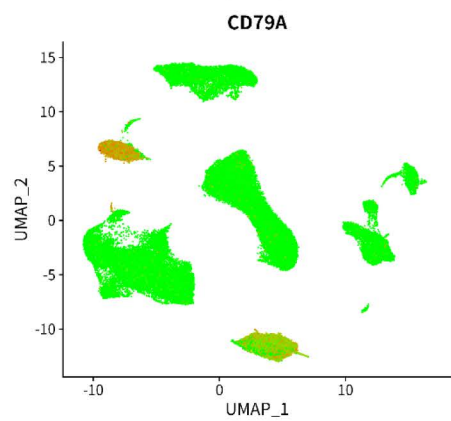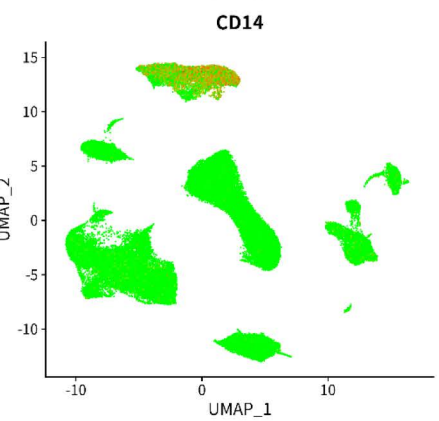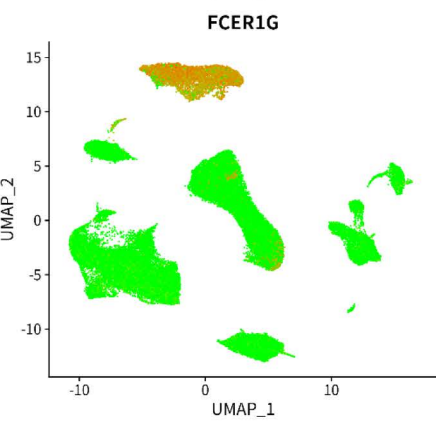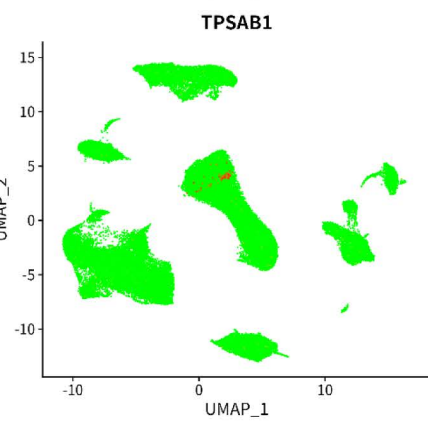

Supplement: Supplementary 2 — Figure S2: UMAP plots of markers for each cell type in CRC. [file 3781091.f2.pdf]
