# Supplementary material for: Cancer-Associated Fibroblast Risk Model for Prediction of Colorectal Carcinoma Prognosis and Therapeutic Responses
Source: Mediators Inflamm. 2023 Apr 25;2023:3781091. doi: 10.1155/2023/3781091 (PMC10154103; doi:10.1155/2023/3781091)
Supplement: Supplementary 3 — Supplementary Table 1: the primer information of genes. [file 3781091.f3.pdf]

Table S1

ID	sequence (5' to 3')
ZNF532-F	GCGTTTAAGTCTGCCCCAAG
ZNF532-R	GTGTTGGTCAAAGTGGCGAT
COLEC12-F	GCATGGAAACATCTCGCCAAA
COLEC12-R	TCTGTAATCTCACGAAGTTGCTG
GAPDH-F	ACAACTTTGGTATCGTGGAAGG
GAPDH-R	GCCATCACGCCACAGTTTC
